# Three-dimensional microarchitecture of the human placental villous tree in health and disease

**DOI:** 10.3389/fcell.2025.1639740

**Published:** 2025-09-10

**Authors:** Nirav Barapatre, Hans-Georg Frank

**Affiliations:** ^1^ Department of Anatomy II, Anatomische Anstalt, LMU Munich, Munich, Germany

**Keywords:** placenta, FGR, preeclampisa, GDM, villous tree, trophoblast, 3D microscopic imaging, stereological analyses

## Abstract

**Background:**

Placental dysfunction plays a central role in pregnancy complications such as fetal growth restriction (FGR), preeclampsia (PE), and gestational diabetes mellitus (GDM). Recent advances in 3D microscopy and stereological analysis have revealed microanatomical changes not detectable by conventional histology.

**Objective:**

To summarise key morphological and cellular alterations in the human placenta across FGR, PE, and GDM, with a focus on architecture of the villous tree, proliferative trophoblast dynamics, and sex-specific adaptations.

**Methods:**

A synthesis of quantitative 3D histological studies was undertaken, focusing on villous compartment volumes, trophoblast proliferation markers (PCNA), nuclear distribution patterns, and branching indices in placentas from affected and control pregnancies.

**Results:**

FGR placentas exhibit central loss of contractile villi (C-villi), increased syncytial nuclear density, and abolished sexual dimorphism. In PE, peripheral villous volume (NC-villi) is reduced, with marked increased proliferation of trophoblast in female placentas and disrupted nuclear spacing. GDM placentas show a global reduction in villous branching and altered proliferative dynamics of villous trophoblast, particularly in females, already in the absence of placental macrosomia.

**Conclusion:**

Despite distinct clinical profiles, FGR, PE, and GDM exhibit specific yet partially overlapping placental microstructural pathologies, characterised by trophoblast dysregulation and sex-specific adaptations. These findings underscore the significance of fetal sex and quantitative three-dimensional morphometry in advancing our understanding of placental disease mechanisms.

## 1 Introduction

### 1.1 Placental histopathologic lesions in obstetric syndromes

Pregnancies complicated by preeclampsia (PE), fetal growth restriction (FGR), or maternal diabetes (GDM) exhibit various patterns of placental histopathological lesions. These lesions include infarcts, fibrinoid deposition, syncytial knots, thrombosis, villitis, chorangiosis, villous dysmaturity and overall abnormalities in placental size and were recently standardized by the Amsterdam Criteria (Khong et al., 2016). While there is no single pathognomonic lesion for any of these syndromes, certain patterns are considered to be suggestive of specific maternal-fetal conditions (Table 1).

**TABLE 1:** Key placental lesions in preeclampsia (PE), fetal growth restriction (FGR), and gestational diabetes mellitus (GDM).

Lesion	PE	FGR	GDM
Infarcts	Seen in ∼ 40%, rising to 60%–70% in early-onset PE (Sebire et al., 2005)	24%–42% depending on gestational age and comorbid hypertension (Salafia et al., 1992; Veerbeek et al., 2014)	Relatively infrequent ( ∼ 10%) (Huynh et al., 2015; Byrne et al., 2025; Lai et al., 2024)
Fibrinoid Deposition	Common, including acute atherosis (Redline, 2015)	Increased fibrin-type deposition associated with vascular dysfunction (Park et al., 2002)	Seen in poorly controlled GDM, often with villous immaturity (Huynh et al., 2015; Daskalakis et al., 2008)
Syncytial Knotting	Very common in early-onset PE (up to 94%) (Heazell et al., 2007)	Also frequent (86%–93%) (Veerbeek et al., 2014)	Typically absent or reduced; often with villous immaturity instead (Lai et al., 2024; Daskalakis et al., 2008)
Thrombosis	Less prominent; maternal vascular malperfusion predominates (Redline, 2015)	Increased in early-onset severe FGR (up to 32%) (Veerbeek et al., 2014)	Not consistently reported; generally not a key feature (Huynh et al., 2015)
Villitis	Not consistently elevated (Park et al., 2002)	May affect up to 30% of placentas (Salafia et al., 1992)	Typically not increased (Huynh et al., 2015)
Placental Size	Often reduced; < 10th percentile in 50% of early-onset PE (Veerbeek et al., 2014)	Average placental weight ∼ 20% lower (Salafia et al., 1992)	Placentomegaly common in poorly controlled GDM (Huynh et al., 2015)

#### 1.1.1 Placental infarcts

Placental infarcts represent areas of ischemic necrosis and are frequently seen in disorders associated with maternal vascular malperfusion (MVM). In PE, approximately 40% of placentas show infarcts, rising to 60%–70% in early-onset, severe cases (Sebire et al., 2005). In FGR, infarcts are also common, seen in 24%–42% of cases depending on severity, gestational age, and coexisting hypertension (Salafia et al., 1992; Veerbeek et al., 2014). In contrast, infarcts were described with an occurrence rate of about 10% and thus are less common in GDM placentas. (Huynh et al., 2015; Byrne et al., 2025; Lai et al., 2024).

#### 1.1.2 Fibrinoid deposition

Fibrinoid deposition, including perivillous fibrin and fibrinoid necrosis of vessel walls (acute atherosis), is a hallmark of placental malperfusion. In early-onset PE, acute atherosis is frequently observed, reflecting impaired spiral artery remodelling (Redline, 2015). In FGR, perivillous fibrin deposition is also significantly increased. A histopathological study by Park et al. found that fibrinoid changes occurred more frequently in FGR placentas compared to controls, highlighting disrupted utero-placental blood flow as a likely contributor (Park et al., 2002). In pregnancies complicated by diabetes mellitus, especially when glycaemic control is suboptimal, placental changes such as villous immaturity, chorangiosis, and fibrinoid necrosis are frequently reported. These lesions reflect both metabolic stress and adaptive angiogenesis. Systematic reviews and histopathological studies confirm that such features are more pronounced in poorly controlled GDM (Huynh et al., 2015; Daskalakis et al., 2008).

#### 1.1.3 Syncytial knotting

An increase in syncytial knots (Tenney–Parker changes) is a hallmark of villous maturation under hypoxic stress. In PE, nearly all early-onset cases demonstrate this lesion, with prevalence reaching 94% (Heazell et al., 2007). FGR placentas also frequently exhibit syncytial knotting (86%–93%) (Veerbeek et al., 2014). In contrast, GDM is associated with delayed villous maturation and is not usually associated with the same degree of increased syncytial knots as PE and FGR (Lai et al., 2024; Daskalakis et al., 2008).

#### 1.1.4 Thrombosis

Thrombotic lesions can occur in both maternal and fetal vessels. In PE, fetal vessel thrombosis is less commonly observed, with MVM being the predominant histopathologic pattern (Redline, 2015). Conversely, in early-onset severe FGR, fetal thrombosis is more prevalent, occurring in up to 32% of cases (Veerbeek et al., 2014). In diabetic pregnancies, placental abnormalities such as villous immaturity, delayed maturation, and increased angiogenesis are common, but thrombotic lesions are reported inconsistently and are not considered a major histopathological feature (Huynh et al., 2015).

#### 1.1.5 Villitis

Chronic villitis of unknown etiology (VUE) is an immunologic lesion with variable prevalence. It is not consistently elevated in PE (Park et al., 2002), but may be more common in FGR, affecting approximately 30% of placentas (Salafia et al., 1992). In diabetic pregnancies, VUE is not a predominant histopathological feature (Huynh et al., 2015).

#### 1.1.6 Placental size abnormalities

Placental size is frequently reduced in PE and FGR due to vascular insufficiency combined with preterm birth. In early-onset PE, placental weight is below the age-adjusted 10th percentile in 50% of cases (Veerbeek et al., 2014). FGR placentas are, on average, approximately 20% smaller compared to those from uncomplicated pregnancies (Salafia et al., 1992). Conversely, diabetic pregnancies, particularly those with poor glycemic control, often exhibit placentomegaly as part of fetoplacental macrosomia (Huynh et al., 2015).

### 1.2 The diagnostic strength of placental histopathology

Placental histopathology reveals lesions common to both normal and pathological pregnancies. As summarised in Table 1, many findings—such as infarcts, fibrinoid deposition, and syncytial knots—occur in normal placentas and are increased to a variable degree across various obstetric syndromes including PE, FGR, and GDM (Veerbeek et al., 2014; Salafia et al., 1992; Huynh et al., 2015; Byrne et al., 2025; Lai et al., 2024). While they may support clinical impressions, these lesions lack specificity and often provide only circumstantial evidence. Histological interpretation must therefore consider clinical context, gestational age, and possible therapeutic interventions. For instance, reduced placental weight in PE may reflect both the disease itself and medically indicated preterm delivery (Sebire et al., 2005). The true diagnostic strength of placental histopathology lies in the identification of infections. Acute chorioamnionitis, characterised by neutrophilic infiltration of membranes and cord, can be reliably diagnosed microscopically and is of high clinical relevance (Redline, 2015). In such cases, histology offers clarity where clinical signs may be subtle or ambiguous.

In summary, while its value in distinguishing villous or vascular and metabolic syndromes is limited, histopathology remains essential for diagnosing intrauterine infection. PE, FGR, and GDM are syndromes which are diagnosed primarily by clinical, but not by histopathologic parameters. For histopathologists and clinicians, this is a challenging situation. Efforts were therefore made to arrive at more focused and, especially, quantitative morphometric evaluations of placental structure to identify specific histological core correlates of the pathogenesis of the main obstetric syndromes.

## 2 Quantitative microscopy of the human placenta is challenging

Conventional histopathology, as defined by the Amsterdam criteria (Khong et al., 2016), is typically qualitative and subjective, i.e., based on observer interpretation. This approach primarily focuses on analysing the symptomatic endpoints of placental processes, which are presumed to originate from alterations in trophoblast biology and/or villous maturation. The analysis of causal, pathogenetic driving processes such as villous maturation relies on stromal evaluation of villous profiles in two-dimensional thin histological sections, which are categorized as “terminal villi”, “intermediate villi”, and “stem villi”, and are assigned to various regions of the villous tree in the delivered placenta (Demir et al., 1997; Kohnen et al., 1996; Sen et al., 1979; Kaufmann et al., 1979).

The validity of this method for quantitative—and possibly also qualitative—assessment of the villous tree has recently been questioned in a detailed study examining the relevance of two-dimensional analysis of villous trees for understanding their true three-dimensional structure (Haeussner et al., 2015). Particularly in the context of quantitatively assessing villous maturation, the traditional classification into three villous types in term placentas is no longer recommended. Instead, it has been proposed (Haeussner et al., 2015) to classify villi through the identification of myofibroblast markers such as γ -smooth muscle actin ( γ -SMA), using an immunohistochemical approach. γ -SMA marks myofibroblasts located within the perivascular contractile sheath of the villous tree (for review see Krantz and Parker (1963); Graf et al. (1992); Graf et al. (1994); Graf et al. (1995); Graf et al. (1997); Kohnen et al. (1995); Kohnen et al. (1996)) and enables a binary classification into two villous types: γ -SMA-positive villi (contractile villi, or C-villi) and villi lacking perivascular contractile myofibroblasts ( γ -SMA-negative villi, non-contractile villi, or NC-villi). This method eliminates the need for complex analysis of villous stroma in order to categorise villi into three types. The presence or absence of the marker allows a straightforward, observer-independent classification into two villous types (C-villi or NC-villi).

In addition to the challenges faced in analysing the three-dimensional villous tree using two-dimensional sections, conventional histopathology provides no direct access to the villous trophoblast, the crucial epithelial layer at the villous surface. Syncytial knots are often interpreted as indicators of syncytial status, but they represent a complex histological epiphenomenon, resulting from the sectioning process. Syncytial knots, defined as aggregations of syncytiotrophoblastic nuclei at the surface of histologic profiles of villi, have long been regarded as indicators of trophoblast maturation or degeneration, particularly in the context of placental ageing and pathologies such as PE (Tenney and Parker, 1940; Jones and Fox, 1977). However, a growing body of morphological and ultrastructural studies suggests that many of these knots are artefacts of histological sectioning, especially in term placentas where the villous surface becomes highly branched and convoluted (Burton, 1986; Cantle et al., 1987; Küstermann, 1981). Serial sectioning and three-dimensional reconstructions have demonstrated that apparent nuclear aggregates and syncytial bridges often result from tangential cuts through villous protrusions rather than representing true proliferative or apoptotic structures (Kaufmann et al., 1987; Wepler, 1990). More recent molecular and immunohistochemical analyses support a distinction between transcriptionally inactive, oxidatively damaged nuclei (true knots) and section-induced artefacts containing active nuclei (Fogarty et al., 2013). Thus, while some syncytial knots may reflect physiological nuclear senescence, the majority seen in histological sections likely represent interpretive artefacts (Burton and Jones, 2009; Roland et al., 2016; Loukeris et al., 2010). Though dealing with the syncytial surface and its properties, histopathology of syncytial knots is more an epiphenomenon of sectioning of trophoblast rather than a specific reflection of the status of villous trophoblast. Structural complexity (tortuosity and branching of the villous tree, thickness of the trophoblast layer), section thickness, type of histological technique (ultrathin, semithin, formalin-fixed and paraffin embedded, cryostate sectioning), and status of the trophoblast itself (senescence, true knots) are all contributing to the epiphenomenon called “syncytial knotting”. Lacking specificity for a specific pathogenetic process, functional interpretations of increased syncytial knotting are challenging.

### 2.1 Classical stereological studies of placental villi

Early quantitative placental morphology research relied on Stereology, in which randomly sampled, haematoxylin–eosin (HE)-stained, 2D histological sections are analysed to infer 3D structure of the sample (Mayhew, 2006). For example, FGR placentas showed significantly reduced volumes of all villous types along with smaller exchange surface areas (Mayhew et al., 2003). Such findings established a baseline understanding that FGR is associated with an “impoverished” villous tree in terms of bulk structure.

However, classical Stereology still depended on HE-stained 2D histological sections and on skilled human observers to identify the three classical villous types. Moreover, many aspects of villous 3D architecture—such as branching angles, connectivity, and spatial organisation—could principally not be assessed from 2D sections (Haeussner et al., 2016).

### 2.2 Limitations of 2D histology and observer variability

By the mid-2010s, researchers began critically evaluating the reliability of these 2D histology-based classifications. Haeussner et al. tested whether identifying villous types on 2D sections correlates with the villus’s actual position in the 3D placental tree (Haeussner et al., 2015). They found high inter-observer variability and poor correlation with actual 3D positions. This study demonstrated that the classical approach was inconsistent and often inaccurate, highlighting the need for more objective criteria and genuine three-dimensional analysis (Haeussner et al., 2015; Haeussner et al., 2016). The present review focuses on advanced three-dimensional morphological techniques that have been applied to human placentas since these fundamental challenges were first articulated.

## 3 Comparative 3D morphology of villous tree architecture in FGR, PE, and GDM: methods and key findings

### 3.1 Methodological framework: stereology, immunohistochemistry-guided stereology, and 3D microscopy

Stereology is an excellent method for obtaining three-dimensional data of organs, including the placenta, from microscopic thin sections. It is a multi-step procedure designed to ensure that every part of the placenta has an equal chance of being placed under the microscope and getting analysed. The essential principle of Stereology lies in random sampling throughout all stages of histological tissue sampling, tissue preparation, and tissue sectioning. By adhering strictly to random processing, the results can be considered representative of the entire organ (Howard and Reed, 2004).

The application of Stereology to the placenta was pioneered, recommended, and refined primarily through the work of Terry Mayhew, among others (Mayhew et al., 1984; Mayhew et al., 1986; Mayhew and Burton, 1988; Mayhew et al., 1990; Mayhew et al., 1994; Mayhew and Wadrop, 1994; Jackson et al., 1987). To distinguish between different parts of the villous tree, HE sections were traditionally used. The stromal core of villous profiles were then classified as one of three histological villous types: terminal villi, intermediate villi, or stem villi (Demir et al., 1997; Kohnen et al., 1996; Sen et al., 1979; Kaufmann et al., 1979). This approach remained the standard for villous typing until it was challenged for being observer-dependent and subject to considerable inter-observer variability (Haeussner et al., 2015). Such variability inevitably led to inconsistencies in the accurate and consistent allocation of stereological findings to specific villous subtypes.

**FIGURE 1:**
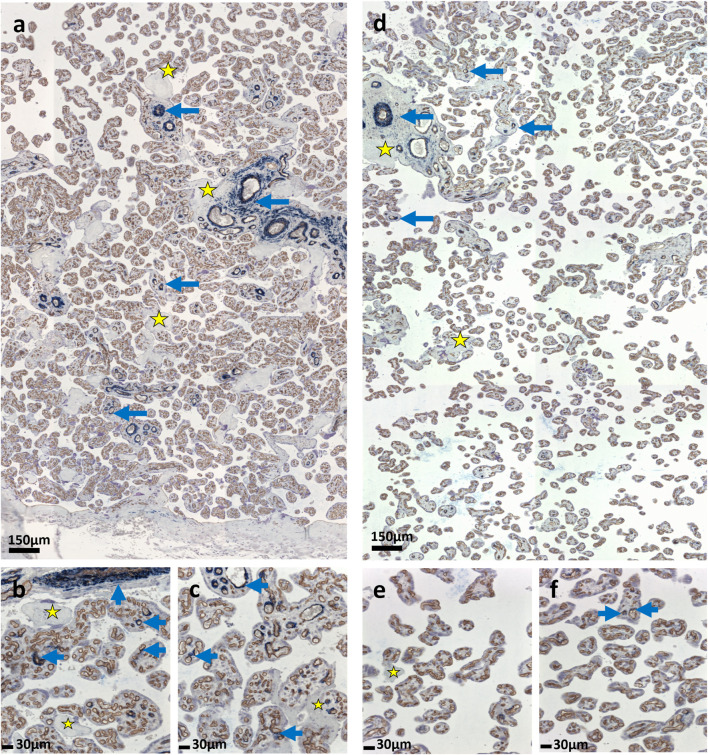
IHC guided Stereology. **(a–f)** The figure shows a representative immunohistochemical double labelling of sections of villous tissue. The γ -sm-actin is labelled by dark blue staining. It is a marker of perivascular myofibroblasts and the perivascular contractile sheath of placental villi. Brown reaction product relates to CD34-detection to mark fetal endothelial cells. The latter assists in the assessment of perivascular location of γ -sm-actin detection as shown by blue arrows at two different magnification scales. **(a–c)** show a control placenta, whereas **(d–f)** show a placenta with fetal growth restriction (FGR). The villous tree is thus compartmentalized and quantified in contractile and non-contractile parts based on the presence or absence of myofibroblasts. The yellow asterisks show perivillous fibrinoid depositions. In FGR **(d–f)** the presence of villous profiles with myofibroblasts is less than in the control placenta **(a–c)**.

Subsequent studies sought to address this limitation by employing immunohistochemical markers to identify whether the perivascular stroma of villous profiles was positive or negative for γ -SMA (e.g., Barapatre et al. (2021); Barapatre et al. (2024)), a modification which had already been recommended by Haeussner et al. (2015). This immunohistochemical characterization allowed villous profiles to be classified into just two categories: C-villi and NC-villi. Notably, this classification does not correspond exactly to the traditional histological classifications of terminal, intermediate, or stem villi. Most of the studies reviewed here employ such immunohistochemistry-guided Stereology of the placenta (IHC-guided Stereology, Figure 1). IHC-guided Stereology has also been extended through the inclusion of second-order stereological parameters, particularly the branching index. This dimensionless value represents the percentage of concave villous surfaces observed in villous profiles. It is interpreted as a proxy for branching points, as concave villous surfaces in such profiles are predominantly associated with sectioning through branching regions rather than through straight segments of villi.

**FIGURE 2:**
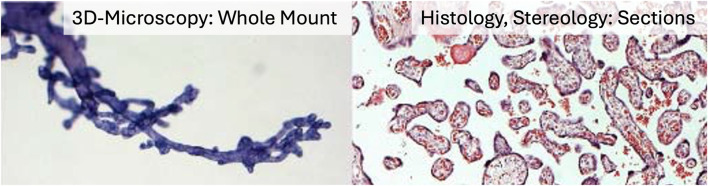
Microscopic approaches of 3D microscopy versus Stereology. The figure illustrates the principal differences between the three-dimensional techniques of Stereology and 3D microscopy. On the left, a whole-mount preparation of a peripheral tip of the villous tree is shown. Villous diameters, branching angles, and trophoblast nuclei are directly visible with appropriate magnification and can be recorded digitally using 3D microscopy. On the right, an HE-stained thin section of villous tissue is shown. Three-dimensional parameters such as villous volumes, branching indices, and diffusion distances can be obtained by analysing such thin histological sections using Stereology.

**FIGURE 3:**
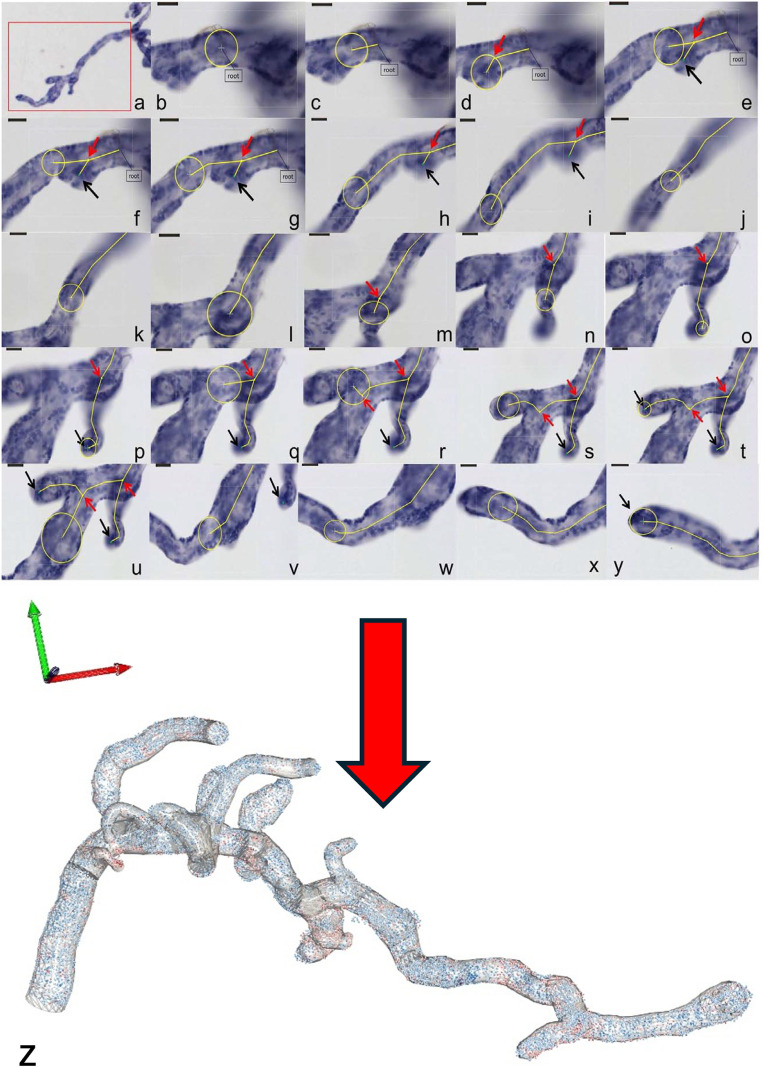
3D microscopy Workflow. **(a–y)** The figure shows a representative workflow of villous quantification by 3D microscopy and **(z)** a 3D reconstruction of the measured villous tree together with the topographic position of each individual trophoblast cell nuclei (in blue for PCNA-negative cell nuclei, in red for PCNA-positive cell nuclei). The villi are measured by tracing a sphere (yellow circles), whose diameter is adjusted continuously to the villous width. The centre line (yellow lines) shows the movement of the sphere through the villi. The small red arrows indicate the branching points. Not shown in the workflow **(a–y)** is the possibility of placing a marker on each of the trophoblast nuclei at high magnification, which records its 3D coordinates and, hence, its topographical position on the villous surface **(z)**. From the latter data, branch-associated or mean surface densities of trophoblast nuclei and nearest neighbour distances between trophoblast nuclei can be calculated.

In contrast, the recently introduced method of three-dimensional (3D) microscopy (Haeussner et al., 2014; Haeussner et al., 2017) uses whole-mount preparations of small peripheral placental villous bushes, thereby eliminating the need for sectioning (Figure 2). The innovative section-free microscopic approach of 3D microscopy to the villous tree is illustrated in Figure 2. It allows direct recognition of branching points and of whole villi—rather than partial profiles—including branching angles, diameters, lengths, and surface areas (Figure 3a–z; Table 2). At higher magnification, the nuclei of individual villous trophoblast cells can be mapped directly onto the villous surface. Immunohistochemical staining, particularly of villous trophoblast, is feasible with such preparations. Software originally developed for tracing dendritic trees in neurons is applied to generate *in silico* reconstructions of peripheral branches via a digitised camera lucida procedure (Figure 3a–z). While 3D microscopy complements Stereology and provides unique data, it focuses specifically on peripheral branches, and its findings cannot be considered representative of the entire villous tree.

**TABLE 2:** Key features of various approaches to quantitative 3D-analysis of the structure of human villous trophoblast and the human placental villous tree.

Approach	Material	Typical endpoints	Comments
Stereology	HE-stained sections	Volumes, Surfaces, maternofetal Diffusion Distance	Uses conventional villous typing by HE-based assessment of villous stroma (terminal villi, intermediate villi, and stem villi). Stereological approaches have a general statistical approach which allows generalisation to the whole placental villous tree
IHC-guided Stereology	Sections, detection of γ -sm-actin	Volumes, Surfaces, maternofetal Diffusion Distance, Branching Index	Villous typing based on profiles of C-villi (with perivascular γ -sm-actin) and NC-villi (no perivascular γ -sm-actin). Stereological approaches have a general statistical approach which allows generalisation to the whole placental villous tree
3D microscopy	Whole mount preparations of bushes of peripheral villi, fixed, combined with immunohistochemical detection of proliferating cell nuclear antigen (PCNA)	Branching angles; length, surface area and volume of the last two branch generations of the villous tree; Surface density and neareast neighbor distances of PCNA-positive and PCNA-negative cell nuclei of villous trophoblast	Access to parameters which are out of reach of any section-based approach. Directly generated from and being applical to the most peripheral two branch generations, but not to the more central parts of the villous tree

### 3.2 FGR: structural disruption of villous growth and trophoblast organisation

Multiple studies employing advanced stereological and three-dimensional microscopic techniques have detailed the morphological alterations in placentas from pregnancies complicated by FGR.

A principal observation is that FGR placentas are not merely smaller versions of normal placentas, but instead display distinct structural pathologies. Quantitative analyses show a significant reduction in the volume of C-villi, which include stem villi characterised by perivascular myofibroblasts. In contrast, NC-villi, representing more peripheral branches, exhibit no statistically significant volume reductions. Vessel volumes are markedly reduced in both compartments, indicating compromised vascularisation throughout the villous tree (Barapatre et al., 2021).

**FIGURE 4:**
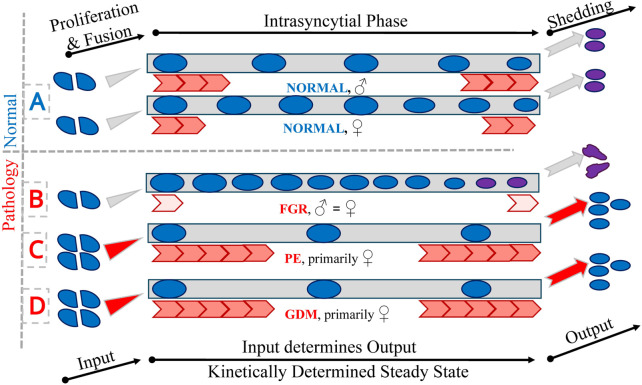
Trophoblast nuclei. The figure presents a schematic representation of trophoblast nuclear passage through the trophoblast layers under normal (A) and pathological conditions (B–D). Blue circles represent trophoblast nuclei undergoing proliferation (PCNA-positive) and subsequent fusion with the syncytiotrophoblast (left). Grey rectangles in the central part of the figure indicate the intrasyncytial phase (PCNA-negative) and the final shedding of trophoblast material into the maternal circulation (right). To maintain a stable and balanced trophoblast system, the input through fusion and the output via shedding must be quantitatively equal, reflecting a kinetically determined dynamic steady state. Red arrows represent qualitatively the kinetics of the intrasyncytial phase, which in turn determines the density of non-proliferative (PCNA-negative) nuclei at the villous surface. This is symbolised by the number of nuclei in the central part of the figure. Panel A shows the physiological process in male and female control placentas, demonstrating the existence of sexual dimorphism under normal conditions. Panels (B–D) depict pathological scenarios: fetal growth restriction (FGR; affecting both sexes equally and thereby abolishing physiological sexual dimorphism), preeclampsia (PE; alterations predominantly observed in female placentas), and gestational diabetes mellitus (GDM; also primarily affecting female placentas), respectively. On the right side of the figure, the outcomes of differing kinetic states are illustrated: in normal conditions, appropriately matured and passivated material is shed (pink circles); in FGR (B), delayed kinetics lead to overaged material being released (depicted as unrounded pink nuclei); in PE and GDM (C,D), accelerated kinetics may result in the premature shedding of material that has not undergone full passivation (depicted as blue nuclei).

At the cellular level, FGR placentas demonstrate a marked increase in the density of PCNA-negative nuclei—representing post-proliferative syncytial nuclei—without a corresponding change in the density of proliferative (PCNA-positive) nuclei. This suggests that trophoblast proliferation is unaltered, while nuclear clearance or syncytial passage time may be impaired (Figure 4). These nuclei tend to accumulate at the villous surface, particularly in peripheral branches, and show increased spatial density, as evidenced by reduced nearest-neighbour distances. These structural reorganisations, undetectable by standard histology, are discernible through three-dimensional microscopy (Haeussner et al., 2017; Barapatre et al., 2019).

Furthermore, the sexual dimorphism normally observed in placental development—manifested by higher syncytial nuclear densities in female placentas and greater inter-nuclear spacing in males—is absent in FGR (Figure 4). This loss of dimorphism suggests a convergent pathological adaptation to growth restriction that overrides physiologically distinct developmental pathways. In normal pregnancies, such dimorphic features are thought to influence fetal vulnerability to intrauterine stress and subsequent postnatal health outcomes (Barapatre et al., 2019).

Branching complexity is also significantly altered in FGR placentas. Using novel 3D reconstruction methods, studies have shown that branching angles and hierarchical organisation of the villous tree correlate with fetoplacental weight ratios, a clinical indicator of placental efficiency. In FGR, this complexity is reduced, implying not only quantitative insufficiency but also a qualitatively aberrant developmental trajectory (Haeussner et al., 2014).

Together, these findings characterise FGR placentation as a condition involving disrupted villous growth, altered syncytial structure, and impaired nuclear dynamics. Sexual dimorphism is effectively neutralised, and the implementation of high-resolution 3D techniques provides a mechanistic insight into the impaired exchange function and increased perinatal risk associated with FGR.

### 3.3 PE: peripheral volume loss and sex-dependent trophoblast changes

A detailed stereological and 3D-microscopy-based investigation of placentas from late-onset PE, stratified by fetal sex, has revealed a complex pattern of trophoblast remodelling (Barapatre et al., 2024). A key observation is the significantly increased proliferative activity, evidenced by elevated PCNA-positive nuclear density, in female PE placentas relative to male PE placentas and controls. Concomitantly, a marked reduction in PCNA-negative nuclei in female PE placentas suggests accelerated trophoblast turnover in this group. This dynamic shift in nuclear composition is mirrored by nearest-neighbour density measures, underscoring the robustness of these findings. No such changes are present in male PE placentas, indicating a sex-specific trophoblast response to the preeclamptic environment. The sexual dimorphism observed in nuclear distribution in control placentas is absent in PE, suggesting disrupted trophoblast architecture across both sexes (Figure 4).

In addition to nuclear changes, PE placentas show significant alterations in villous morphology. The volume of non-contractile villi and their intravillous fetal vessels is reduced across both sexes, reflecting compromised exchange capacity. While this reduction is statistically significant in males, female PE placentas show a comparable trend that does not reach significance, implying a subtler but present effect. The branching index of the villous tree is significantly decreased, indicating impaired arborisation, with the reduction more pronounced in females.

These findings demonstrate that PE induces both quantitative and structural changes in the placenta, with clear sexual dimorphism. These alterations distinguish PE from related conditions such as FGR, where such sex-specific structural changes are less evident. The sex-dependent nature of trophoblast and villous adaptations may influence clinical outcomes, including gestational duration, maternal morbidity, and offspring health trajectories.

### 3.4 GDM: global branching reduction and syncytial alteration in females

Placental alterations in well-controlled gestational diabetes mellitus (GDM) have been investigated using integrated stereological and 3D microscopic techniques (Barapatre et al., 2025). Although gross clinical parameters—including gestational age, birth weight, placental weight, and placental-to-birth weight ratios—do not significantly differ between GDM and control pregnancies, distinct microanatomical changes are evident.

A primary finding is a pronounced reduction in the branching index of both C-villi and NC-villi components, indicating a global simplification of the villous tree architecture. This effect appears independent of fetal sex and suggests impaired villous development affecting both early (stem villi) and late (terminal villi) forming structures.

Trophoblast dynamics further reveal sex-dependent effects (Figure 4). GDM placentas, particularly those of female fetuses, show increased surface density of PCNA-positive nuclei and decreased density of PCNA-negative nuclei. These changes are associated with reduced inter-nuclear distances among proliferative nuclei and increased spacing among non-proliferative nuclei—hallmarks of heightened turnover and abbreviated syncytial residence. Such imbalance likely promotes excess shedding of trophoblast material into maternal circulation, consistent with elevated placenta-derived exosome levels reported in GDM.

While overall villous and vascular volumes remain unaltered, subtle changes such as decreased standard deviation of diffusion distances in NC-villi are observed in females, suggesting functional compensation. These adaptations, though subclinical, highlight the capacity of the placenta to structurally accommodate metabolic stress. The predominance of changes in female placentas mirrors patterns seen in PE (Figure 4) and underscores the critical role of fetal sex in shaping placental resilience.

In conclusion, even in the absence of macrosomia or overt pathology, GDM induces significant trophoblast and villous changes. These alterations may represent an early, sex-specific adaptive response to metabolic dysregulation, with potential implications for the increased incidence of comorbid conditions such as PE.

## 4 Synthesis: morphological signatures across syndromes

• FGR is chiefly characterised by a loss of central villous volume (C-villi, but not NC-villi) and reduced villous vascularisation. Together, these alterations may contribute to the increased fetoplacental resistance characteristic of FGR. The branching index indicates diminished arborisation throughout the villous tree. These changes are accompanied by normal trophoblast proliferation and an accumulation of post-proliferative trophoblast nuclei at the villous surface. The typical sexual dimorphism of villous trophoblast is replaced by a uniform, FGR-associated pattern.

• PE is marked by a reduction in peripheral villous volume (NC-villi) and a general loss of arborisation across the villous tree. Villous trophoblast shows enhanced proliferation, as indicated by increased density of PCNA-positive trophoblast nuclei at the villous surface. In contrast to FGR, the density of post-proliferative trophoblast is reduced—predominantly in placentas from female fetuses.

• GDM is defined by a uniform and markedly pronounced reduction in branching complexity, more severe than in either FGR or PE. Villous tree volumes remain comparable to control placentas (in the absence of fetal or placental macrosomia). The branching index is reduced across all compartments of the villous tree, showing no evidence of sexual dimorphism. These alterations were observed in well-controlled GDM without clinically detectable macrosomia. Villous trophoblast findings mirrored those in PE: increased density of proliferating trophoblast nuclei alongside reduced density of post-proliferative nuclei—again, predominantly in placentas from female foetuses, while male placentas remained unaffected.

The core findings summarised above go beyond prior knowledge generated through immunohistochemistry-guided Stereology and 3D microscopy. This advance is largely due to the methodological benefits provided by these modern approaches in comparison to traditional design-based Stereology on HE-sections without whole-mount 3D microscopy.

### 4.1 Immunohistochemistry-guided stereology and 3D microscopy

Classifying placental villi presents a morphological challenge that has been mitigated through the use of immunohistochemical markers, notably γ -smooth muscle actin ( γ -SMA). This technique enables a binary distinction between “contractile” ( γ -SMA-positive) and “non-contractile” ( γ -SMA-negative) villi, reflecting their position within the branching hierarchy of the villous tree (Buehlmeyer et al., 2019; Barapatre et al., 2021; Kohnen et al., 1995; Kohnen et al., 1996; Graf et al., 1997). This innovation enhances objectivity in villous classification and reduces observer variability associated with HE-based typing (Haeussner et al., 2015). While total villous volume reduction in FGR was already known (Mayhew et al., 2003), it is now possible to localise this loss to the C-villi, sparing the NC-villi.

This stereological toolkit is effectively complemented by whole-mount 3D microscopy. Although this technique is limited to the analysis of peripheral villous branches, it remains the sole light microscopy method that enables a detailed, three-dimensional morphological analysis. Whole-mount specimens extend up to several hundred micrometres in depth and are analysed through a computer-assisted camera lucida method that digitally reconstructs a 3D model of the villous tree. Quantitative parameters such as branching angles, diameters, surface areas, lengths, and volumes can be extracted and spatially assigned to terminal and preterminal positions. Trophoblast phenotyping in these peripheral regions is achieved via immunohistochemical cell cycle markers. Proliferative trophoblast (PCNA-positive) and post-proliferative (PCNA-negative) compartments can be distinguished, allowing further calculations of nuclear surface densities and inter-nuclear distances—metrics not accessible via traditional histology.

Recent innovations in 3D microscopy, including confocal imaging, have further transformed placental analysis. These technologies allow direct visualisation and quantification of villous architecture—including branching angles, tortuosity, and connectivity—that cannot be captured through 2D approaches (Haeussner et al., 2016). These metrics provide critical insights into placental function, particularly under pathological conditions.

### 4.2 The villous branching index

The villous branching index (BI), formerly known as the Concavity Index (CI), can be assessed on standard HE sections, although this precludes association with C- or NC-villi. The method hinges on the premise that branches between villous nodes exhibit rounded profiles. Upon random histological sectioning, these profiles present as convex, straight, or concave. Only branching points yield concave profiles. BI is a second-order stereological measure indicating the percentage of surface area comprising concave villous profiles. A higher BI reflects a greater density of villous branching points (Tables 3, 4; Barapatre et al. (2024); Barapatre et al. (2021)).

**TABLE 3:** Summary of morphological and cellular alterations in placentas from pregnancies affected by fetal growth restriction (FGR), preeclampsia (PE), and gestational diabetes mellitus (GDM), based on IHC-guided Stereology and 3D microscopy. Findings were drawn from Barapatre et al. (2021), Barapatre et al. (2019), Haeussner et al. (2014), Haeussner et al. (2017), Barapatre et al. (2024) (PE) and Barapatre et al. (2025) (GDM). Highlighted features include affected villous compartments, branching patterns, trophoblast kinetics, and sex-specific differences.

Feature	FGR	PE	GDM
Affected Villous Compartment	C-villi (central loss)	NC-villi (peripheral volume loss)	C- and NC-villi (global reduction in arborisation)
Branching Index	Decreased (sex-independent)	Decreased (marked in females)	Decreased (sex-independent)
Trophoblast Proliferation	Unchanged; increased PCNA-negative nuclei (nuclear accumulation)	Increased in females; reduced PCNA-negative nuclei (accelerated turnover)	Increased in females; reduced PCNA-negative nuclei (enhanced turnover)
Nuclear Distribution	Elevated PCNA-negative nuclear density; reduced spacing (syncytial congestion)	Female: compressed syncytial spacing; Male: unchanged	Female: proliferative clustering and immature syncytium; Male: unchanged
Vascular Volume	Reduced in both C- and NC-villi	Reduced in NC-villi (both sexes)	Unchanged
Sex-Specific Findings	Dimorphism abolished in FGR	Present; changes more prominent in females	Present; changes restricted to females

**TABLE 4:** Comparison of selected key studies on placental villous structure. Data on Preeclampsia (PE), fetal growth restriction (FGR), and gestational diabetes mellitus (GDM) are shown. BI is the branching index.

Study (Year)	Methods	Key findings
Mayhew et al. (2003)	Stereology	Reduced villous volume across all villous types in FGR
Haeussner et al. (2015)	Histology and Stereology: Validation of Villous typing	High observer variability, not recommended for quantitative purposes
Haeussner et al. (2016)	3D-Microscopy (confocal)	Altered branching angles in FGR
Buehlmeyer et al. (2019)	IHC-guided Stereology	C-villi volume correlates with placental weight in normal placentas
Barapatre et al. (2021)	IHC-guided Stereology	Selective reduction of C-villi volume in FGR
Barapatre et al. (2024)	IHC-guided Stereology + 3D microscopy	Sexual dimorphism of trophoblast, reduced BI in PE
Barapatre et al. (2025)	IHC-guided Stereology + 3D microscopy	Sexual dimorphism of trophoblast, reduced BI in GDM

FGR, PE, and GDM are associated with a lower BI than healthy controls, indicating reduced branching complexity.

• FGR Placentas: These placentas exhibit a significantly reduced frequency of concave villous profiles, indicating diminished branching. The villous tree is developmentally altered, not merely miniaturised. No sex differences were observed in BI in FGR placentas (Barapatre et al., 2021).

• Preeclamptic Placentas: PE placentas, particularly those from late-onset cases, show a decreased BI relative to healthyS controls. Both contractile (central) and non-contractile (peripheral) villi display fewer concave surfaces. This decrease is more pronounced in placentas from female foetuses, despite largely unaltered total villous volumes (Barapatre et al., 2024).

• GDM Placentas: Well-controlled GDM placentas, even in the absence of macrosomia, reveal a marked, sex-independent reduction in BI, with no significant differences in overall placental volume or weight compared to controls (Barapatre et al., 2025).

### 4.3 Synthesis: scientific and clinical impact and outlook

Often referred to as the “mirror of the prenatal period,” the placental functional microarchitecture is now revealed in unprecedented detail through modern histological techniques. Traditional Stereology and histopathology laid the groundwork, but were limited in resolution and specificity. Recent research by Haeussner, Barapatre, Buehlmeyer, Lahti-Pulkkinen and colleagues (Haeussner et al., 2014; Haeussner et al., 2015; Haeussner et al., 2017; Barapatre et al., 2021; Barapatre et al., 2019; Barapatre et al., 2024; Lahti-Pulkkinen et al., 2018) illustrates how the integration of immunohistochemistry and 3D microscopy refines our understanding. By delineating central versus peripheral villi, one can identify specific compartments affected by pathology. Quantification of nuclear and branching characteristics in 3D exposes regulatory phenomena previously undetectable in 2D. Stratifying morphological data by sex or linking placental metrics to offspring outcomes further embeds placental pathology within personalised medicine.

Importantly, these studies do not merely describe phenotypes but offer mechanistic insights. For instance, the identification of reduced volume of the more centrally located C-villi in FGR redirects attention to early placental development, implicating mesenchymal and vasculogenic defects (Tables 3, 4; Barapatre et al. (2021)). The emergence of sex-specific placental responses in PE suggests hormonal or genetic modulation, potentially informing risk assessment (Barapatre et al., 2024). Correlations between placental structure and child neurodevelopment (Lahti-Pulkkinen et al., 2018) imply that placental structural features could serve as a proxy for intrauterine conditions, encouraging examination even in pregnancies without overt complications.

From a methodological perspective, these findings support the practical feasibility of integrating quantitative histology into routine diagnostics. Modern computing enables scalable application of systematic sampling, point counting, and 3D reconstruction. With reasonable sample sizes, rigorous and reproducible quantitative analyses are achievable. Routine pathology may soon incorporate selected immunohistochemical stains (e.g., γ -SMA, PCNA) and appropriate, possibly simplified tools to objectively quantify placental function.

In summary, while current histological standards (e.g., Amsterdam criteria, Khong et al. (2016)) provide a vital framework for placental evaluation, the innovations discussed herald a new era of “enhanced placental histology” (Figure 4; Table 3). Here, subtle microstructural alterations with macroscale implications can be detected and quantified, advancing both scientific understanding and clinical practice. Future integration of these methods promises to bridge the remaining gaps between placental structure, function, and long-term health outcomes.
